# [Retracted] Engulfment of platelets delays endothelial cell aging via girdin and its phosphorylation

**DOI:** 10.3892/ijmm.2026.5958

**Published:** 2026-08-17

**Authors:** Yong Lan, Yongjun Li, Dajun Li, Peng Li, Jiyang Wang, Yongpeng Diao, Guodong Ye, Yangfang Li

Int J Mol Med 42: 988-997, 2018; DOI: 10.3892/ijmm.2018.3685

Following the publication of this paper, it was drawn to the Editor's attention by a concerned reader that the cellular images shown in Fig. 7A on p. 995 were strikingly similar to data in a paper that had previously been published in the journal *Molecular Cancer* that was written by different authors at different research institutes. Furthermore, an independent analysis of the data in this paper performed by the Editorial Office revealed that flow cytometric data in Fig. 2A, the cell migration and invasion assay data in Fig. 3A and D and the control western blot data in Fig. 5A were also strikingly similar to data that had either previously been published in articles written by different authors at different research institutes, or which were submitted for publication at around the same time.

Given that the abovementioned data had already been published before the receipt of this paper at *International Journal of Molecular Medicine*, the Editor has decided that this paper should be retracted from the Journal. The authors were asked for an explanation to account for these concerns, but the Editorial Office did not receive a reply. The Editor apologizes to the readership for any inconvenience caused.

